# 4,4′-Dimethoxy-2,2′-[1,1′-(propane-1,3-diyldinitrilo)diethylidyne]diphenol

**DOI:** 10.1107/S1600536808023738

**Published:** 2008-08-06

**Authors:** Hoong-Kun Fun, Reza Kia

**Affiliations:** a X-ray Crystallography Unit, School of Physics, Universiti Sains Malaysia, 11800 USM, Penang, Malaysia

## Abstract

In the crystal structure, the title Schiff base compound, C_21_H_26_N_2_O_4_, has twofold rotation symmetry. The imino group is coplanar with the aromatic ring. An intra­molecular O—H⋯N hydrogen bond forms a six- membered ring, producing an *S*(6) ring motif. The two benzene rings are almost perpendicular to each other, making a dihedral angle of 85.00 (2)°. The meth­oxy group is approximately coplanar with the benzene ring, with a C—O—C—C torsion angle of 2.34 (12)°. Neighbouring mol­ecules are linked together by weak inter­molecular C—H⋯O hydrogen bonds and a C—H⋯π inter­action, forming a sheet parallel to the *ab* plane. The mol­ecules also adopt a zigzag arrangement along the *c* axis.

## Related literature

For bond-length data, see: Allen *et al.* (1987 ▶). For hydrogen-bond motifs, see: Bernstein *et al.* (1995 ▶). For information on Schiff base ligands and complexes, and their applications, see, for example: Fun, Kargar & Kia (2008 ▶); Fun, Kia & Kargar (2008 ▶); Fun, Mirkhani *et al.* (2008**a* ▶,b* ▶); Calligaris & Randaccio (1987 ▶); Casellato & Vigato (1977 ▶); Kia, Mirkhani, Kalman & Deak (2007 ▶); Kia, Mirkhani, Harkema & van Hummel (2007 ▶); Pal *et al.* (2005 ▶); Reglinski *et al.* (2004 ▶); Hou *et al.* (2001 ▶); Ren *et al.* (2002 ▶).
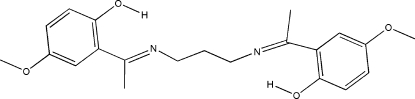


## Experimental

### 

#### Crystal data

C_21_H_26_N_2_O_4_
*M* *_r_* = 370.44
Monoclinic, 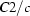

*a* = 12.8042 (2) Å
*b* = 5.0508 (1) Å
*c* = 28.6019 (6) Å
β = 93.109 (2)°
*V* = 1847.00 (6) Å^3^
*Z* = 4
Mo *K*α radiation
μ = 0.09 mm^−1^
*T* = 100.0 (1) K
0.47 × 0.44 × 0.29 mm

#### Data collection

Bruker SMART APEXII CCD area-detector diffractometer
Absorption correction: multi-scan (**SADABS**; Bruker, 2005 ▶) *T*_min_ = 0.884, *T*_max_ = 0.974
34801 measured reflections
5229 independent reflections
4304 reflections with *I* > 2σ(*I*)
*R*_int_ = 0.031

#### Refinement

*R*[*F* ^2^ > 2σ(*F* ^2^)] = 0.057
*wR*(*F* ^2^) = 0.148
*S* = 1.11
5229 reflections
125 parameters
H-atom parameters constrained
Δρ_max_ = 0.45 e Å^−3^
Δρ_min_ = −0.28 e Å^−3^

### 

Data collection: *APEX2* (Bruker, 2005 ▶); cell refinement: *APEX2*; data reduction: *SAINT* (Bruker, 2005 ▶); program(s) used to solve structure: *SHELXTL* (Sheldrick, 2008 ▶); program(s) used to refine structure: *SHELXTL*; molecular graphics: *SHELXTL*; software used to prepare material for publication: *SHELXTL*, *PLATON* (Spek, 2003 ▶) and *PARST95* (Nardelli, 1995 ▶).

## Supplementary Material

Crystal structure: contains datablocks global, I. DOI: 10.1107/S1600536808023738/is2318sup1.cif

Structure factors: contains datablocks I. DOI: 10.1107/S1600536808023738/is2318Isup2.hkl

Additional supplementary materials:  crystallographic information; 3D view; checkCIF report

## Figures and Tables

**Table 1:** Hydrogen-bond geometry (Å, °)

*D*—H⋯*A*	*D*—H	H⋯*A*	*D*⋯*A*	*D*—H⋯*A*
O1—H1*O*1⋯N1	0.97	1.62	2.5241 (10)	153
C11—H11*A*⋯O1^i^	0.96	2.53	3.4448 (12)	160
C11—H11*B*⋯O1^ii^	0.96	2.53	3.4360 (12)	157
C10—H10*C*⋯*Cg*1^iii^	0.96	2.68	3.5224 (10)	147

Symmetry codes: (i) 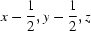; (ii) 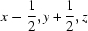; (iii) 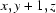. *Cg*1 is the centroid of the C1–C6 benzene ring.

**Fig. 1.**
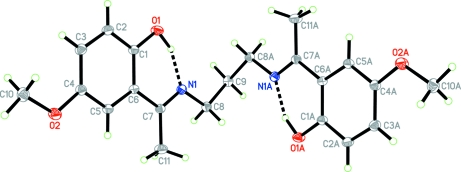
The molecular structure of (I) with atom labels and 50% probability ellipsoids for non-H atoms. The suffix A corresponds to symmetry code (-x + 1, y, -z + 1/2).

**Fig. 2.**
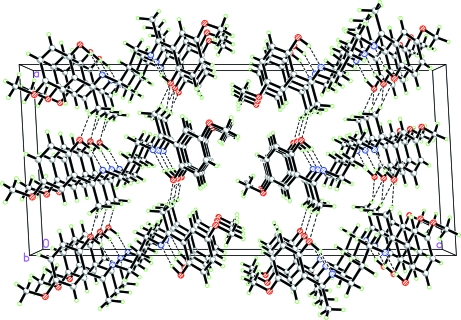
The crystal packing, showing stacking of molecules down the b axis. Intramolecular and intermolecular interactions are shown as dashed lines.

**Fig. 3.**
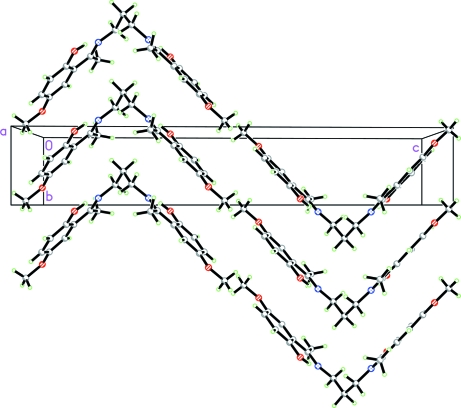
The crystal packing, showing zigzag arrangement of molecules along the c axis.

## supplementary crystallographic information

### Comment

The condensation of primary amines with carbonyl compounds yields Schiff base (Casellato & Vigato, 1977) that are still now regarded as one of the most potential group of chelators for facile preparations of metallo-organic hybrid materials. In the past two decades, the synthesis, structure and properties of Schiff base complexes have stimulated much interest for their noteworthy contributions in single molecule-based magnetism, materials science, catalysis of many reactions like carbonylation, hydroformylation, reduction, oxidation, epoxidation and hydrolysis, etc (Kia, Mirkhani, Kalman & Deak, 2007; Kia, Mirkhani, Harkema & van Hummel, 2007; Pal et al., 2005; Reglinski et al., 2004; Hou et al., 2001; Ren et al., 2002). This is due to the fact that Schiff bases offer opportunities for inducing substrate chirality, tuning the metal-centered electronic factor and enhancing the solubility and stability of either homogeneous or heterogeneous catalysts. Only a relatively small number of free Schiff base ligands have been characterized (Calligaris & Randaccio, 1987). As an extension of our work (Fun, Kargar & Kia, 2008; Fun, Kia & Kargar, 2008; Fun et al.,  2008a,b) on the structural characterization of Schiff base compounds, the title compound (I), is reported here.

The molecule of the title compound, (I), has a crystallographic twofold rotation symmetry (Fig. 1). The bond lengths and angles are within normal ranges (Allen et al.,1987). The asymmetric unit of the compound is composed of one-half of the molecule. An intramolecular O—H···N hydrogen bond forms a six-membered ring, producing an S(6) ring motif (Bernstein et al. 1995). The two benzene rings are almost perpendicular to each other with a dihedral angle of 85.00 (2)°. The methoxy group is coplanar with the benzene ring, with the C10–O2–C4–C3 torsion angle of 2.34 (12)°. In the crystal structure neighbouring molecules are linked together by weak intermolecular C—H···O hydrogen bonds and a C—H···π interaction to form a sheet parallel to the ab plane (Fig. 2). These molecules also adopt a zigzag arrangement along the c axis (Fig. 3).

### Experimental

The synthetic method has been described earlier (Reglinski *et al.*, 2004). Single crystals suitable for *X*-ray diffraction were obtained by evaporation of an ethanol solution at room temperature.

### Refinement

H atom bound to O1 was located from a difference Fourier map and refined as riding, with *U*_iso_(H) = 1.5*U*_eq_(O). Other H atoms were positioned geometrically (C—H = 0.93 – 0.96 Å) and refined using a riding model. A rotating-group model was applied for the methyl groups.

### Crystal data

**Table:**

C_21_H_26_N_2_O_4_	*F*_000_ = 792
*M**_r_* = 370.44	*D*_x_ = 1.332 Mg m^−^^3^
Monoclinic, *C*2/*c*	Mo *K*α radiation λ = 0.71073 Å
Hall symbol: -C 2yc	Cell parameters from 9900 reflections
*a* = 12.8042 (2) Å	θ = 2.9–38.4º
*b* = 5.0508 (1) Å	µ = 0.09 mm^−^^1^
*c* = 28.6019 (6) Å	*T* = 100.0 (1) K
β = 93.109 (2)º	Block, yellow
*V* = 1847.00 (6) Å^3^	0.47 × 0.44 × 0.29 mm
*Z* = 4	

### Data collection

**Table:**

Bruker SMART APEXII CCD area-detector diffractometer	5229 independent reflections
Radiation source: fine-focus sealed tube	4304 reflections with *I* > 2σ(*I*)
Monochromator: graphite	*R*_int_ = 0.031
*T* = 100.0(1) K	θ_max_ = 38.8º
φ and ω scans	θ_min_ = 2.9º
Absorption correction: multi-scan(*SADABS*; Bruker, 2005)	*h* = −22→18
*T*_min_ = 0.884, *T*_max_ = 0.974	*k* = −8→8
34801 measured reflections	*l* = −48→50

### Refinement

**Table:**

Refinement on *F*^2^	Secondary atom site location: difference Fourier map
Least-squares matrix: full	Hydrogen site location: inferred from neighbouring sites
*R*[*F*^2^ > 2σ(*F*^2^)] = 0.057	H-atom parameters constrained
*wR*(*F*^2^) = 0.148	*w* = 1/[σ^2^(*F*_o_^2^) + (0.0571*P*)^2^ + 1.5152*P*] where *P* = (*F*_o_^2^ + 2*F*_c_^2^)/3
*S* = 1.11	(Δ/σ)_max_ < 0.001
5229 reflections	Δρ_max_ = 0.45 e Å^−^^3^
125 parameters	Δρ_min_ = −0.28 e Å^−^^3^
Primary atom site location: structure-invariant direct methods	Extinction correction: none

### Special details

**Table:**

Experimental. The low-temperature data was collected with the Oxford Cyrosystem Cobra low-temperature attachment.
Geometry. All e.s.d.'s (except the e.s.d. in the dihedral angle between two l.s. planes) are estimated using the full covariance matrix. The cell e.s.d.'s are taken into account individually in the estimation of e.s.d.'s in distances, angles and torsion angles; correlations between e.s.d.'s in cell parameters are only used when they are defined by crystal symmetry. An approximate (isotropic) treatment of cell e.s.d.'s is used for estimating e.s.d.'s involving l.s. planes.
Refinement. Refinement of *F*^2^ against ALL reflections. The weighted *R*-factor *wR* and goodness of fit *S* are based on *F*^2^, conventional *R*-factors *R* are based on *F*, with *F* set to zero for negative *F*^2^. The threshold expression of *F*^2^ > σ(*F*^2^) is used only for calculating *R*-factors(gt) *etc*. and is not relevant to the choice of reflections for refinement. *R*-factors based on *F*^2^ are statistically about twice as large as those based on *F*, and *R*- factors based on ALL data will be even larger.

### Fractional atomic coordinates and isotropic or equivalent isotropic displacement parameters (Å^2^)

**Table:**

	*x*	*y*	*z*	*U*_iso_*/*U*_eq_	
O1	0.59948 (5)	0.57780 (14)	0.14698 (2)	0.01790 (13)	
H1O1	0.5565	0.4795	0.1680	0.027*	
O2	0.32409 (5)	1.27531 (14)	0.05669 (2)	0.01928 (13)	
N1	0.44774 (6)	0.41178 (14)	0.19154 (2)	0.01406 (12)	
C1	0.53083 (6)	0.74582 (16)	0.12471 (3)	0.01383 (13)	
C2	0.56814 (6)	0.91899 (18)	0.09166 (3)	0.01619 (14)	
H2A	0.6385	0.9140	0.0851	0.019*	
C3	0.50202 (7)	1.09968 (17)	0.06815 (3)	0.01633 (14)	
H3A	0.5281	1.2150	0.0463	0.020*	
C4	0.39649 (6)	1.10628 (16)	0.07764 (3)	0.01411 (13)	
C5	0.35797 (6)	0.93301 (16)	0.11033 (3)	0.01378 (13)	
H5A	0.2873	0.9380	0.1162	0.017*	
C6	0.42331 (6)	0.75110 (15)	0.13460 (3)	0.01242 (13)	
C7	0.38248 (6)	0.57157 (16)	0.17015 (3)	0.01281 (13)	
C8	0.41119 (7)	0.23770 (17)	0.22797 (3)	0.01519 (14)	
H8A	0.3572	0.1216	0.2145	0.018*	
H8B	0.3807	0.3436	0.2520	0.018*	
C9	0.5000	0.0715 (2)	0.2500	0.01590 (19)	
H9	0.4651	−0.0665	0.2746	0.019*	
C10	0.35993 (8)	1.44822 (19)	0.02170 (3)	0.01964 (16)	
H10A	0.3025	1.5528	0.0090	0.029*	
H10B	0.3883	1.3457	−0.0029	0.029*	
H10C	0.4131	1.5628	0.0353	0.029*	
C11	0.26922 (7)	0.57899 (19)	0.18136 (4)	0.01984 (16)	
H11A	0.2370	0.4123	0.1733	0.030*	
H11B	0.2343	0.7180	0.1638	0.030*	
H11C	0.2639	0.6114	0.2142	0.030*	

### Atomic displacement parameters (Å^2^)

**Table:**

	*U*^11^	*U*^22^	*U*^33^	*U*^12^	*U*^13^	*U*^23^
O1	0.0124 (2)	0.0187 (3)	0.0228 (3)	0.0024 (2)	0.0023 (2)	0.0063 (2)
O2	0.0159 (3)	0.0187 (3)	0.0233 (3)	0.0031 (2)	0.0012 (2)	0.0076 (2)
N1	0.0143 (3)	0.0140 (3)	0.0139 (3)	−0.0006 (2)	0.0013 (2)	0.0014 (2)
C1	0.0119 (3)	0.0142 (3)	0.0155 (3)	0.0007 (2)	0.0015 (2)	0.0006 (2)
C2	0.0130 (3)	0.0177 (3)	0.0181 (3)	0.0002 (3)	0.0032 (2)	0.0024 (3)
C3	0.0160 (3)	0.0162 (3)	0.0170 (3)	−0.0008 (3)	0.0028 (3)	0.0027 (3)
C4	0.0137 (3)	0.0132 (3)	0.0154 (3)	0.0011 (2)	0.0008 (2)	0.0010 (2)
C5	0.0120 (3)	0.0139 (3)	0.0155 (3)	0.0005 (2)	0.0015 (2)	0.0005 (2)
C6	0.0114 (3)	0.0124 (3)	0.0135 (3)	0.0001 (2)	0.0018 (2)	−0.0001 (2)
C7	0.0122 (3)	0.0125 (3)	0.0137 (3)	−0.0008 (2)	0.0013 (2)	−0.0009 (2)
C8	0.0156 (3)	0.0152 (3)	0.0147 (3)	−0.0021 (2)	0.0011 (2)	0.0011 (3)
C9	0.0191 (5)	0.0125 (4)	0.0161 (5)	0.000	0.0000 (4)	0.000
C10	0.0229 (4)	0.0173 (3)	0.0186 (4)	0.0016 (3)	0.0005 (3)	0.0046 (3)
C11	0.0134 (3)	0.0215 (4)	0.0250 (4)	0.0007 (3)	0.0045 (3)	0.0063 (3)

### Geometric parameters (Å, °)

**Table:**

O1—C1	1.3556 (10)	C5—H5A	0.9300
O1—H1O1	0.9728	C6—C7	1.4787 (11)
O2—C4	1.3736 (10)	C7—C11	1.5025 (11)
O2—C10	1.4230 (11)	C8—C9	1.5221 (11)
N1—C7	1.2917 (11)	C8—H8A	0.9700
N1—C8	1.4597 (11)	C8—H8B	0.9700
C1—C2	1.3915 (12)	C9—C8^i^	1.5221 (11)
C1—C6	1.4208 (11)	C9—H9	1.1014
C2—C3	1.3928 (12)	C10—H10A	0.9600
C2—H2A	0.9300	C10—H10B	0.9600
C3—C4	1.3929 (12)	C10—H10C	0.9600
C3—H3A	0.9300	C11—H11A	0.9600
C4—C5	1.3906 (12)	C11—H11B	0.9600
C5—C6	1.4010 (11)	C11—H11C	0.9600
			
C1—O1—H1O1	103.6	C6—C7—C11	120.84 (7)
C4—O2—C10	116.90 (7)	N1—C8—C9	111.49 (6)
C7—N1—C8	119.36 (7)	N1—C8—H8A	109.3
O1—C1—C2	118.35 (7)	C9—C8—H8A	109.3
O1—C1—C6	121.93 (7)	N1—C8—H8B	109.3
C2—C1—C6	119.71 (7)	C9—C8—H8B	109.3
C1—C2—C3	121.18 (8)	H8A—C8—H8B	108.0
C1—C2—H2A	119.4	C8—C9—C8^i^	113.08 (10)
C3—C2—H2A	119.4	C8—C9—H9	107.1
C2—C3—C4	119.50 (8)	C8^i^—C9—H9	113.8
C2—C3—H3A	120.3	O2—C10—H10A	109.5
C4—C3—H3A	120.3	O2—C10—H10B	109.5
O2—C4—C5	115.40 (7)	H10A—C10—H10B	109.5
O2—C4—C3	124.67 (7)	O2—C10—H10C	109.5
C5—C4—C3	119.94 (7)	H10A—C10—H10C	109.5
C4—C5—C6	121.46 (7)	H10B—C10—H10C	109.5
C4—C5—H5A	119.3	C7—C11—H11A	109.5
C6—C5—H5A	119.3	C7—C11—H11B	109.5
C5—C6—C1	118.21 (7)	H11A—C11—H11B	109.5
C5—C6—C7	121.26 (7)	C7—C11—H11C	109.5
C1—C6—C7	120.52 (7)	H11A—C11—H11C	109.5
N1—C7—C6	117.71 (7)	H11B—C11—H11C	109.5
N1—C7—C11	121.44 (7)		
			
O1—C1—C2—C3	−178.88 (8)	C2—C1—C6—C5	0.02 (12)
C6—C1—C2—C3	0.44 (13)	O1—C1—C6—C7	0.25 (12)
C1—C2—C3—C4	−0.42 (13)	C2—C1—C6—C7	−179.04 (8)
C10—O2—C4—C5	−177.58 (8)	C8—N1—C7—C6	177.96 (7)
C10—O2—C4—C3	2.34 (13)	C8—N1—C7—C11	−1.07 (12)
C2—C3—C4—O2	−179.99 (8)	C5—C6—C7—N1	−179.06 (8)
C2—C3—C4—C5	−0.07 (13)	C1—C6—C7—N1	−0.02 (11)
O2—C4—C5—C6	−179.53 (7)	C5—C6—C7—C11	−0.02 (12)
C3—C4—C5—C6	0.54 (13)	C1—C6—C7—C11	179.01 (8)
C4—C5—C6—C1	−0.51 (12)	C7—N1—C8—C9	−178.18 (7)
C4—C5—C6—C7	178.54 (7)	N1—C8—C9—C8^i^	58.96 (5)
O1—C1—C6—C5	179.32 (8)		

Symmetry codes: (i) −*x*+1, *y*, −*z*+1/2.

### Hydrogen-bond geometry (Å, °)

**Table:**

*D*—H···*A*	*D*—H	H···*A*	*D*···*A*	*D*—H···*A*
O1—H1O1···N1	0.97	1.62	2.5241 (10)	153
C11—H11A···O1^ii^	0.96	2.53	3.4448 (12)	160
C11—H11B···O1^iii^	0.96	2.53	3.4360 (12)	157
C10—H10C···Cg1^iv^	0.96	2.68	3.5224 (10)	147

Symmetry codes: (ii) *x*−1/2, *y*−1/2, *z*; (iii) *x*−1/2, *y*+1/2, *z*; (iv) *x*, *y*+1, *z*.
